# Sonographic follow-up after endoscopic carpal tunnel release for severe carpal tunnel syndrome: a one-year neuroanatomical prospective observational study

**DOI:** 10.1186/s12891-019-2548-6

**Published:** 2019-04-09

**Authors:** Miao Li, Jue Jiang, Qi Zhou, Chen Zhang

**Affiliations:** 1 Department of Ultrasound, The Second Affiliated Hospital of Xi’an Jiaotong University, Xi’an Shaanxi, 710004 People’s Republic of China; 2 The First Department of Orthopaedics, The Second Affiliated Hospital of Xi’an Jiaotong University, Xi’an Shaanxi, 710004 People’s Republic of China

**Keywords:** Carpal tunnel syndrome, Carpal tunnel release, Median nerve, High frequency ultrasound, Arthroscopy

## Abstract

**Background:**

Endoscopic carpal tunnel release (ECTR) has been gradually adopted for the treatment of severe carpal tunnel syndrome (CTS). However, perioperative assessment of neuroanatomical parameters of median nerve, which are important determinant of median nerve recovery, has rarely been reported. This one-year prospective study aimed to investigate the natural history of the neuroanatomical morphology of the median nerve after ECTR in severe CTS patients by high-frequency ultrasonography and assess the ability of neuroanatomical measures to quantify morphological recovery of the median nerve after ECTR.

**Methods:**

This study recruited 31 patients (44 wrists) with a definitive diagnosis of severe CTS and underwent ECTR operation. The edema length (EL) of median nerve from the inlet of the carpal tunnel to the proximal wrist was detected on long axis imaging plane and the anteroposterior diameter (D) and cross-sectional area (CSA) at the inlet of the carpal tunnel on short axis imaging plane were detected by high frequency ultrasound. All these metrics were detected at 3 days before surgery and at the 2nd week, 4th week, 3rd month, 6th month and 12th month after surgery separately.

**Results:**

There was no significant difference of each parameter between the 2-week postoperative (1.914 ± 0.598 cm in EL, 0.258 ± 0.039 cm in D and 0.138 ± 0.015 cm^2^ in CSA) and 3-days preoperative time points (*P*-EL =0.250; *P*-D = 0.125; *P*-CSA =0.712). From the fourth week to the third month after surgery, the parameters quickly improved. The EL (0.715 ± 0.209 cm), D (0.225 ± 0.017 cm) and CSA (0.117 ± 0.012 cm^2^) at the 3- month postoperative time points were more reduced than at the fourth week after surgery (*P*-EL < 0.001; *P*-D = 0.038; *P*-CSA =0.014). Thereafter, the neurological anatomy parameters recovered slowly. By the 12-month postoperative time points, the three parameters were neuroanatomically close to normal. Compared to the control group in D (0.213 ± 0.005 cm), there was no difference at the 12-month time point (0.214 ± 0.009 cm, *P* = 0.939). However, the difference in EL (0.098 ± 0.030 cm vs. 0.016 ± 0.011 cm) and CSA (0.103 ± 0.008 cm^2^ vs. 0.073 ± 0.005 cm^2^) between patients and healthy volunteers at the 12-month time point still existed (*P*-EL < 0.001; *P*-CSA < 0.001).

**Conclusions:**

Neuroanatomical parameters were gradually improved after ECTR surgery. The best time for US follow up is at 3-month postoperative time point for patients who do not show clinical improvement, since at this time the change is the greatest for most CTS patients. This study has been registered in Chinese Clinical Trial Registry: ChiCTR-ROC-17014068 (retrospectively registered 20-12-2017).

## Background

Carpal tunnel syndrome (CTS) is a common peripheral nerve disorder [1]. CTS of long duration or with severe symptoms, especially with abnormal electrophysiological findings, should be treated surgically [2]. Successful surgical management depends on release of the transverse carpal ligament, whether through open or minimally invasive approaches. Scar adhesions after conventional open carpal tunnel release (OCTR) operation easily lead to recurrence and affect the appearance of the wrists [3]. Endoscopic carpal tunnel release (ECTR), with the advantage of reduced trauma, low recurrence rate and quick recovery of wrist function, has been gradually adopted for the treatment of severe CTS [4, 5]. However, a meta-analysis of a randomized controlled trial showed no differences in the overall complication rate, subjective satisfaction, time to return to work, postoperative grip and pinch strength, or operative time between the two operation groups [6]. The pros and cons of the two operations are still inconclusive. Therefore, further objective clinical study needed to be performed.

At present, many researchers [7–10] have reported a large number of clinical advantages or disadvantages of ECTR surgery, which have mainly focused on the convenience of surgical operation, safety, complications incidence, postoperative function of hand, pain relief rate, extent of neurophysiological recovery, and rehabilitation time. However, the neuroanatomical parameters of the median nerve, which are an important determinant of median nerve recovery, have rarely been studied.

With the development of ultrasound imaging, including the application of high-frequency probes (18 MHz) and the improvement of scanning methods, ultrasound scanning can display the median nerve with high resolution and real-time assessment [11, 12]. Previous studies mainly focused on the evaluation of the diagnostic power and value of CTS using ultrasound. Little has been written concerning the analysis of neuroanatomical parameters before and after ECTR. To the best of the authors’ knowledge, only two reports have described the neurophysiological recovery before and after endoscopic intervention in CTS patients. Abicalaf [13] prospectively studied 20 patients with CTS before and at 4, 8, and 12 weeks after surgery and found that there was a progressive reduction in the cross-sectional area (CSA) of the median nerve after intervention. El-Karabaty H [14] used high-resolution ultrasound to quantify the flattening ratio before endoscopic release, as well as 2 weeks and 3 months postoperatively. They concluded that ultrasound is a simple and excellent objective method for visualizing the morphological recovery of the median nerve very early after decompression surgery. As is known, nerve recovery is a long-term process, and 3-month follow-up studies cannot fully reflect the course of nerve recovery during the entire postoperative process. In addition, more clinical evidence on the neuroanatomical parameters of the median nerve after ECTR is needed. This one-year prospective study aimed to investigate the natural history of the neuroanatomical morphology of the median nerve after ECTR in severe CTS patients by high-frequency ultrasonography and assess the ability of neuroanatomical measures to quantify morphological recovery of the median nerve after ECTR.

## Methods

### Participants

**Fig. 1:**
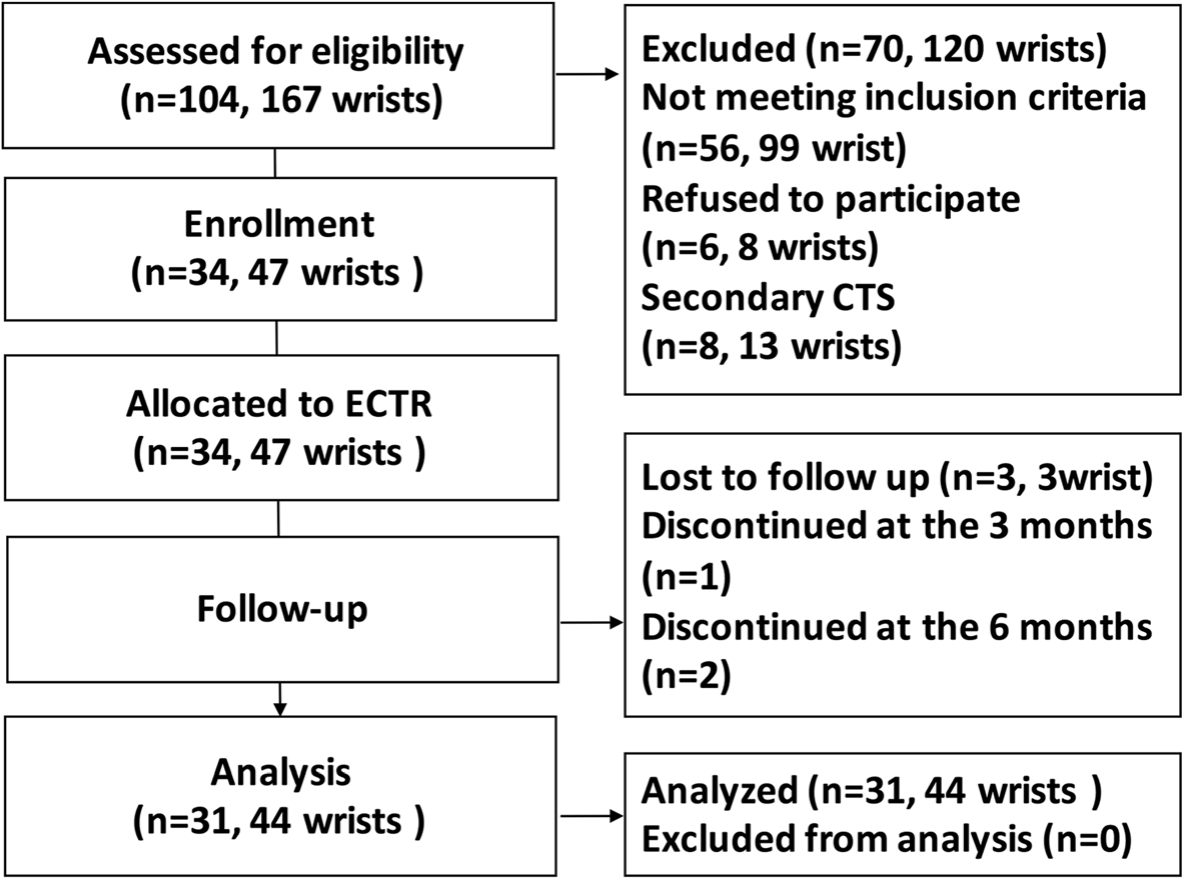
Subject inclusion decision tree

From December 2014 to October 2016, 167 wrists (104 patients) were assessed for eligibility, and 47 wrists (34 patients) met the entry criteria and were included in the study. Three were lost during follow-up. The final prospective analysis included 44 wrists of 31 patients (10 males and 21 females, with age range of 29–68 years; mean, 53.4 ± 8.7 years; and an average duration of CTS diagnosis of 3.4 ± 1.6 year) with a definitive clinical diagnosis of severe idiopathic CTS who underwent electrodiagnostic testing (EDT), high-frequency ultrasonic examination, and ECTR (13 double wrist surgery and 18 single wrist surgery). The controls included 46 wrists of 23 healthy volunteers matched for age and gender who were randomly recruited from healthy individuals at the hospital (8 males and 15 females, with an age range of 30–65 years; mean, 51.46 ± 6.4 years). Both wrists were evaluated in each volunteer of the control group. The following inclusion criteria were used for the patient group: (1) diagnosed as severe idiopathic CTS by clinical symptoms, signs and EDT; (2) underwent ECTR surgery in our hospital (all operations were performed by the same surgeon); (3) provided informed consent. The following exclusion criteria were applied to the patient group: (1) did not complete testing of all the time points or was lost to follow-up; (2) diagnosis of secondary CTS (history of wrist and hand fracture, history of wrist and hand surgery, neurological disorders, thyroid disorders, diabetes, rheumatoid arthritis, gout, pregnant and lactating women, et al). Figure 1 shows a detailed flowchart describing our selection criteria and decision-making process in determining study patients.

### Severe CTS diagnosis

The diagnostic evidence of severe CTS was acquired from clinical symptoms, signs and EDT. The clinical symptoms of severe CTS were classified according to the Gelberman Classification [15] and defined as severe numbness along the distribution of the median nerve of the wrist or hand, obvious thenar muscle atrophy and thumb opposition dysfunction, as well as two-point discrimination over 10 mm. Positive Tinel’s sign, Phalen’s test and Durkan’s test supported the diagnosis. The diagnosis of severe CTS by EDT was classified according to a modified scoring system [16]. The principle criteria of diagnosis of severe CTS are shown in Table 1.

**Table 1:** Diagnostic principles of severe carpal tunnel syndrome

Symptoms	
*Meet the following four symptoms:*	
*•* Severe numbness in the distribution of the median nerve of the wrist or hand;	
*•* Obvious thenar muscle atrophy;	
*•* Thumb opposition dysfunction;	
*•* Two-point discrimination > 10 mm;	
Signs	
*Meet one of the following three signs:*	
*•* Positive Tinel’s sign;	
*•* Positive Phalen’s test;	
*•* Positive Durkan’s test;	
Electrodiagnostic testing	
*Meet the following two electrodiagnostic results:*	
*•* Absence of median sensory nerve action potential (SNAPS) (digit-wrist segment)	
*•* abnormal distal motor latency (DML ≥ 4.2 ms) or absence of motor response;	

### Electrodiagnostic testing

All EDT were performed according to the protocol [17] suggested by the American Association of Electrodiagnostic Medicine recommendations by a same neurologist using a Nicolet-Viking IV electromyograph (Natus Medical Incorporated, CA, USA) and at a standard room temperature of 25 °C. Hand temperature was maintained above 31 °C by applying hot packs. (1) Electromyography (EMG) was performed with a disposable concentric needle electrode inserted into the abductor pollicis brevis (APB) muscle. The spontaneous potential including fibrillation potential and positive phase potential in resting state muscle was documented. (2) Motor nerve conduction studies (NCSs) were recorded by surface electrode from the abductor pollicis brevis (APB) muscle for the median nerve, and separately from the abductor digiti minimi (ADM) muscle for the ulnar nerve. The stimulation points were over the carpal tunnel for the median nerve and the Guyon canal for the ulnar nerve. The distal motor latency (DML), motor nerve conduction velocities (MNCV) and the amplitudes of the compound muscle action potentials (CMAPs) of the median and ulnar nerve were recorded. (3) The sensory nerve conduction velocities (SNCV) and sensory nerve action potential (SNAPS) of the median and ulnar nerve were stimulated from the second digit and fifth digit, respectively, and recorded at the wrist antidromically. The difference of distal sensory latency (DSL) between the median nerve and the ulnar nerve was stimulated from the ring finger and recorded at the wrist antidromically. CTS was accepted as (1) DML ≥ 4.2 ms, MNCV ≤45 m/s, DSL ≥3.6 ms, SNCV ≤40 m/s or difference of DSL ≥ 0.4 ms; (2) ulnar NCSs of the same side were normal [18, 19].

### ECTR surgery

Using well-established methods [20], the surgery was performed under local anaesthesia. Two portals were needed to perform the surgery. A 1–1.5 cm line from the proximal tip of the pisiform was drawn radially. A second line was drawn proximally from the end of this first line 0.5 cm. After that, a third line was drawn from the proximal end of the second line radially at 1 cm that determined the entry portal. For exit portal, the thumb was brought to full abduction. A transverse line was drawn from the distal end of the abducted thumb and a longitudinal line between the 3rd and 4th finger was drawn proximally in the palm surface. These two lines formed a right angle. A line bisecting this right angle was extended 1 cm from the vertex towards the ulna. This established the site of incision for the exit portal. The entry portal was opened, and the fascia was exposed with a blunt dissection and cut. A gap was created between the transverse carpal ligament (TCL) and the ulnar bursa (common flexor sheath of hand) [21] with the help of a curved dissector after the proximal border of the TCL was determined. Then, a slotted cannula was inserted through the entry portal under the TCL. An incision was made at the exit portal and the cannula was extracted. The TCL was visualized with an endoscope and divided with a retrograde knife (ECTRA 2; Smith and Nephew). The completion of the cut was checked by endoscope. The cannula was removed. The portals were closed with nonabsorbable sutures. A compression bandage was applied to control postoperative bleeding and released 30 min after surgery in all patients.

### High-frequency ultrasonic examination

We used a colour Doppler ultrasonic diagnosis apparatus (Asendus, HITACHI, Japan) with an 18 L5 linear array probe. Every patient was examined by the same ultrasound physician, who has musculoskeletal ultrasound experience. All ultrasound examinations were performed as described previously [22], while the subjects were seated facing the examiner. The participants’ arms were extended, and the elbows were flexed at 45 degrees; the forearm was in the supine position, and the wrists rested on a flat surface. Care was taken to examine the wrists in a neutral position and avoid any wrist or hand position that increased the carpal tunnel pressure. The fingers were held in a relaxed position while they were semiflexed. Two-dimensional ultrasound was used for scanning examination of the median nerve. The median nerve was first identified on the long axis for observing the anatomical localization and a continuous boundary trace of the nerve. On the long axis imaging plane, the edema length (EL) of the median nerve was detected and defined as the length of the edema of the median nerve from the inlet of the carpal tunnel (the level of distal wrist crease / proximal end of the pisiform) to the proximal wrist (the level of distal radioulnar joint). Then, the transducer was rotated 90° to obtain a short axis imaging plane for testing the anteroposterior diameter (D) and the CSA of the median nerve at the inlet of the carpal tunnel. All tests were performed 3 days before surgery and at 2 weeks, 4 weeks, 3 months, 6 months and 12 months after surgery. Each patient was examined three times at each follow-up. The mean values of each parameter were defined as the final values for each time point.

### Statistical analysis

Statistical analyses were performed using SPSS 22.0 software (SPSS, Chicago, Illinois). The measurement data were expressed as the mean ± SD. Hypothesis tests were 2-tailed and used a 5% significance level. Linear mixed model analysis was performed to evaluate neuroanatomical changes in the patient groups at different time points and between patients and volunteers at the same time point. All original data were subjected to standard normal conversion first. ID numbers for all participants were distributed and treated as subject variables. Time was treated as a repeated categorical variable. The results of EL, D and CSA were selected as dependent variables. The group and time were modelled as covariates separately.

## Results

### Participants

The demographic data of the participants were in Table 2. There were no significant differences in the age and sex between the patients and control groups (*P*-Age = 0.231; *P*-Sex = 0.864). There were significant differences in the EL, D and CSA between the patients and control groups (*P* < 0.001).

**Table 2:** Demographics and baseline data

Variables	Patients (*n* = 31, 44 wrists)	Control (*n* = 23, 46 wrists)	*P*-value
Age (yr.)	53.4 ± 8.7 (29–68)	51.46 ± 6.4 (30–65)	0.231
Male/Female (*n*)	10 /21	8/15	0.864
Side (M/F, *n*)			
Double sides	13 (4/9)	23(8/15)	
Single side	18 (6/12)	0(0/0)	
Occupation (*n*, %)			
Office employee	13 (41.9)	10 (43.5)	
Factory employee	11 (35.5)	8 (34.8)	
Soldier	1 (3.2)	0 (0)	
Freelance worker	6(19.4)	5(21.7)	
Duration of symptoms (yr.)	3.4 ± 1.6	–	
EL - inlet(cm)	2.073 ± 0.718	0.016 ± 0.011	< 0.001
D - inlet(cm)	0.272 ± 0.045	0.213 ± 0.005	< 0.001
CSA - inlet(cm2)	0.141 ± 0.029	0.073 ± 0.005	< 0.001

### Ultrasound follow-up

#### 2nd week post-surgery follow-up

**Fig. 2:**
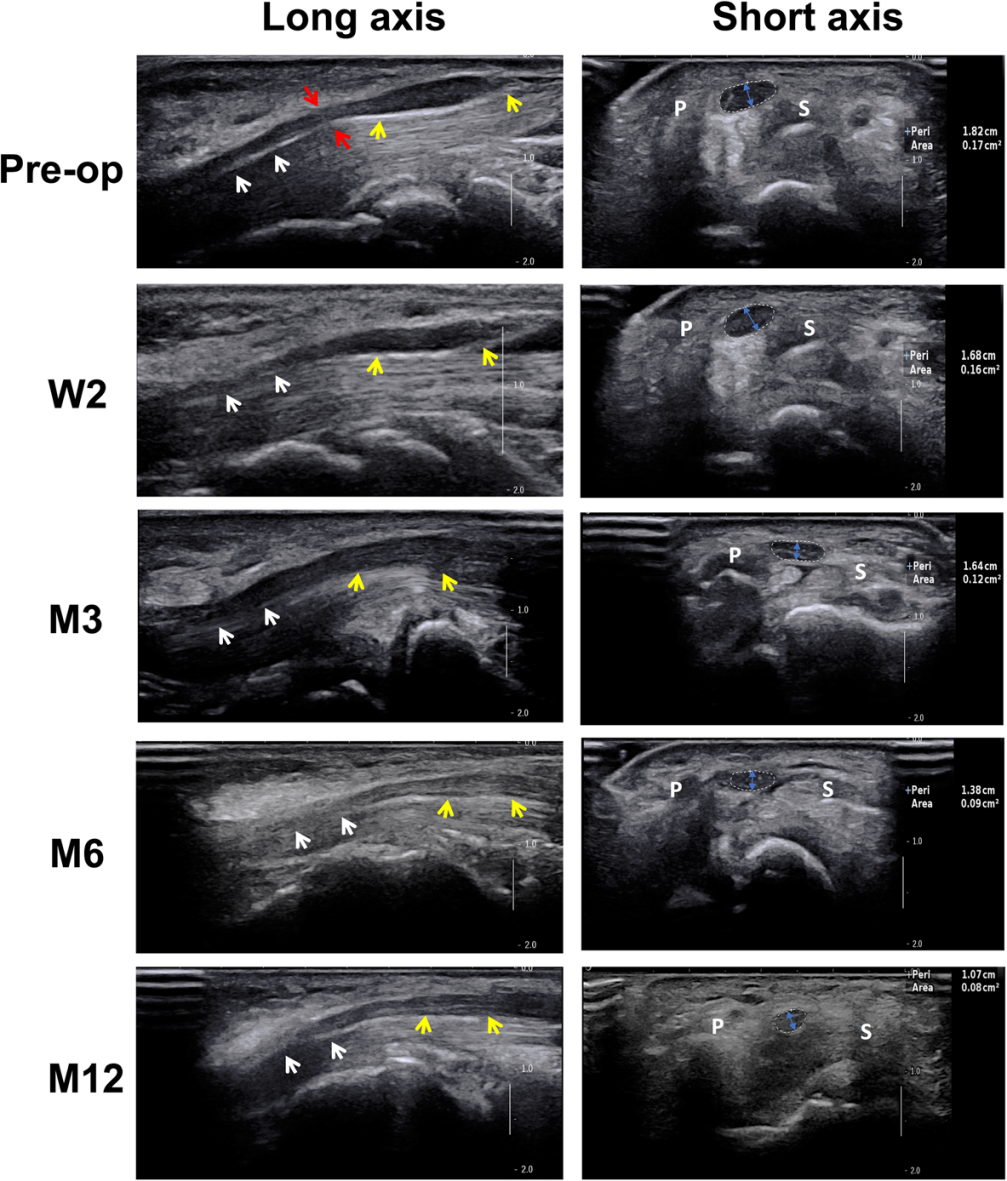
High frequency ultrasonic imaging of carpal tunnel from right hand of a 48-year-old man with severe CTS. The long axis figures of median nerve were showed in left column. The white arrow indicated the median nerve at the distal end of the carpal tunnel, and the yellow arrow indicated the median nerve at the proximal end of the carpal tunnel. Between the both yellow arrow was the edema length (EL) of median nerve. The red arrow pointed at the compression site. Representative figures on the short axis plane at the inlet of the carpal tunnel (the level of distal wrist crease) were showed in the right column. The cross-sectional area (CSA) of the median nerve was delineated by dashed line and the values were showed on the right side. The anteroposterior diameter (D) of median nerve was showed by blue double arrow line. P and S indicated pisiform and scaphoid separately. Pre-op: 3 days pre-surgery; W2: 2 weeks post-surgery; M3: 3 months post-surgery; M6: 6 months post-surgery; M12: 12 months post-surgery

Interval changes of neuroanatomical parameters for the median nerve during the 1-year follow-up are shown in Table 3. Long axis and short axis imaging of the median nerve by high-frequency ultrasonic examination are shown in Fig. 2. At 3 days before surgery, the median nerve was significantly oedematous and thickened at the carpal tunnel entrance. The three parameters of the patient group (2.073 ± 0.718 cm in EL, 0.272 ± 0.045 cm in D and 0.141 ± 0.029 cm^2^ in CSA) were significantly greater than those of the control group (0.016 ± 0.011 cm in EL, *P* < 0.001; 0.213 ± 0.005 cm in D, *P* < 0.001; 0.073 ± 0.005 cm^2^ in CSA, *P* < 0.001). The EL, D and CSA of the patient group improved slightly within 2 weeks after surgery due to postoperative traumatic edema. For each parameter, there was no significant difference between the 2-week postoperative (1.914 ± 0.598 cm in EL, 0.258 ± 0.039 cm in D and 0.138 ± 0.015 cm^2^ in CSA) and 3-days preoperative time points (*P*-EL =0.250; *P*-D = 0.125; *P*-CSA =0.712).

**Table 3:** Interval changes of neuroanatomical parameters for the median nerve (mean ± SD)

Parameter	Patient (*n* = 31, 44 wrists)						Control (*n* = 23, 46 wrists)
	3d pre-surgery	2nd week post-surgery	4th week post-surgery	3rd month post-surgery	6th month post-surgery	12th month post-surgery	
EL, cm	2.073 ± 0.718^#^	1.914 ± 0.598^#^	1.246 ± 0.504*^#^	0.715 ± 0.209*^#^	0.429 ± 0.129*^#^	0.098 ± 0.030*^#^	0.016 ± 0.011
D, cm	0.272 ± 0.045^#^	0.258 ± 0.039^#^	0.237 ± 0.032*^#^	0.225 ± 0.017*^#^	0.219 ± 0.013*^#^	0.214 ± 0.009*	0.213 ± 0.005
CSA, cm^2^	0.141 ± 0.029^#^	0.138 ± 0.015^#^	0.124 ± 0.014*^#^	0.117 ± 0.012*^#^	0.109 ± 0.011*^#^	0.103 ± 0.008*^#^	0.073 ± 0.005

*EL* edema length of median nerve, *D* anteroposterior diameter of median nerve, *CSA* cross-sectional area of median nerve, *SD* standard deviation;

*Statistical comparison with the previous time point for each parameter in patient group, *p* < 0.05;

^#^Statistical comparison with control group, *p* < 0.05

#### 4th week post-surgery follow-up

As postoperative traumatic edema subsided and nerve compression was lifted, the neuroanatomical parameters improved. At the 4-week postoperative time point, the three parameters in the patient group (1.246 ± 0.504 cm in EL, 0.237 ± 0.032 cm in D and 0.124 ± 0.014 cm^2^ in CSA) had improved significantly. For each parameter, there was a significant difference between the 2-week and the 4-week postoperative time points (*P*-EL < 0.001; *P*-D = 0.002; *P*-CSA < 0.001).

#### 3rd month post-surgery follow-up

From the fourth week to the third month after surgery, the parameters of neuroanatomy quickly improved. The EL (0.715 ± 0.209 cm) at 3 months was more reduced than at the fourth week after surgery, and the D (0.225 ± 0.017 cm) and CSA (0.117 ± 0.012 cm^2^) of ​​nerve were significantly reduced at the third month. Compared to the fourth week postoperative time point, there were significant differences of each parameter at the third month postoperative time point (*P*-EL < 0.001; *P*-D = 0.038; *P*-CSA =0.014).

#### 6th month post-surgery follow-up

After 3 months after surgery, the neurological anatomy parameters recovered slowly. However, the neuroanatomical parameters were still gradually improving. For each parameter, there was significant difference between the 6-month (0.429 ± 0.129 cm in EL, 0.219 ± 0.013 cm in D and 0.109 ± 0.011 cm^2^ in CSA) and the 3-month postoperative time points (*P*-EL < 0.001; *P*-D = 0.041; *P*-CSA =0.001).

#### 12th month post-surgery follow-up

Although there were significant differences in EL, D and CSA between the 6-month and the 12-month time points (*P*-EL < 0.001; *P*-D = 0.013; *P*-CSA =0.003), the three parameters were neuroanatomically close to normal. Compared to the control group in D (0.213 ± 0.005 cm), there was no difference at the 12-month time point (0.214 ± 0.009 cm, *P* = 0.939). However, the difference in EL (0.098 ± 0.030 cm vs. 0.016 ± 0.011 cm) and CSA (0.103 ± 0.008 cm^2^ vs. 0.073 ± 0.005 cm^2^) between patients and healthy volunteers at the 12-month time point still existed (*P*-EL < 0.001; *P*-CSA < 0.001).

## Discussion

At present, ECTR surgery has been gradually adopted for the treatment of severe CTS. However, perioperative assessment of neuroanatomical parameters of the median nerve, which are important determinants of median nerve recovery, has rarely been performed. To the best of the authors’ knowledge, this is the first one-year prospective observational study to describe the natural history of the neuroanatomical morphology of the median nerve after ECTR in severe CTS patients by high-frequency ultrasonography. The results from this study provide evidence that there was a progressive reduction in the EL, D and CSA of the median nerve after ECTR. The important findings of this study were that all the neuroanatomical parameters improved significantly between 4 weeks to 3 months after surgery. This finding is highly in accordance with previous NCSs. All these findings indicate that ECTR surgery is a clinically effective treatment for severe CTS and high-frequency ultrasonography is a valuable method in neuroanatomical measures to quantify morphological recovery of the median nerve after ECTR.

In prior studies, [23, 24] most anatomic characterizations of the median nerve in CTS patients showed a flat median nerve at the level of the hook of the hamate (the thickest location of TCL) and a dilative median nerve at the level of the proximal end of the pisiform. At the pisiform near the hook of the hamate (the middle level of the carpal tunnel), the anterior median nerve wall of most CTS patients is relatively weak [25]. Nerve compression at the level of the hook of the hamate resulted in microcirculatory disorders and blocking of the median nerve axoplasmic flow, which results in intrafascicular edema. Prolonged compression causes epineural and endoneural interstitial edema [26], and the CSA of the median nerve to be enlarged at the level of the proximal pisiform. Therefore, enlargement of the CSA of the median nerve at the proximal end of pisiform (carpal tunnel inlet) was the most characteristic morphological change in CTS. We examined the EL, D and CSA of the median nerve and found that thickening of the median nerve at the proximal pisiform and median nerve edema were significant, as demonstrated by more severe clinical symptoms, and an increased disease course. The EL, D and CSA of the median nerves of the preoperative patients were significantly greater than those of the healthy group. Therefore, if the median nerve is released from the compressed carpal tunnel, the median nerve axoplasmic flow should be gradually improved until almost normal. Our findings indicated the neuroanatomical parameters gradually improved postoperatively. From the improvement in the neuroanatomical data, we validated the above pathophysiological process.

Knowledge of the time course and degree of improvement of the median nerve after carpal tunnel release are useful and important parameters for surgeons. There seems to be a consensus that the neurophysiological recovery rate is curvilinear; very high at first and then progressively lower. Borisch [27] prospectively studied 307 patients of CTS and performed a one-year follow-up nerve conduction studies after OCTR surgery. The results indicated that most of the recovery of DML happened during the first 3 months, and the mean DML had not returned to normal by the 3-month postoperative assessment. Rotman [28] evaluated prospectively electrophysiologic parameters using the automated nerve conduction system in patients with CTS both before and after ECTR surgery and found that the majority of the electrophysiologic improvement was realized by 6 weeks, similar to the findings of Pascoe [29], with maximal DML improvement occurring by 3 months. Seror [30] also made the same conclusion that the improvement in SNCV of postoperative carpal tunnel release obtained in the first 2 months after surgery represented 66% of the gain at 1 year. However, the previous studies on the time course and degree of improvement of the median nerve after carpal tunnel release, especially the long-term (6 months to 1 year) prospective studies, mainly relied on the NCSs, and no ultrasonic evaluation of neuroanatomical parameters was performed. The important findings of this study were that all the neuroanatomical parameters were improved significantly during fourth week to third month after surgery, which were highly in accordance with previous NCSs. These findings indicate that surgeons should strengthen postoperative rehabilitation and follow-up of patients during this period. Insufficient CTR or postoperative scar compression may lead to slow improvement of these parameters during this period. Meanwhile, we found that the neuroanatomical parameters were close to normal 1 year after the operation, which suggested the necessity of long-term follow-up after CTR.

In this prospective observational study, the neuroanatomical results, which were from 44 wrists of 31 patients and 46 wrists of 23 healthy volunteers, were repeated measures acquired at different times points and not actually independent from each other. Despite repeated-measures analysis of variance (RM-ANOVA) being a simple and common choice for analysing repeated measures in the clinical trials, there are many restrictions in using this method of analysis. RM-ANOVA depends on a restrictive sphericity assumption [31], which is highly questionable for longitudinal data, because measurements taken closely together are often more correlated than those taken at larger time intervals [32]. Additionally, it cannot handle mis-timed or unbalanced measurements, and the results often require post hoc analyses [33]. Linear mixed model analyses (LMMs) are more amenable to real-world clinical data as opposed to highly controlled experimental study designs. It allows for flexible modelling of the time effect and time-varying covariates. Meanwhile, LMMs permit unbalanced data with greatly different numbers of measurements per subject [33]. Although the implementation and complexity of fitting in LMMs is relatively more difficult than RM-ANOVA, the integration of these model-fitting methods into routine statistical software such as SPSS, SAS and Stata therefore remove a major barrier to applied researchers. We successfully performed a linear mixed model analysis by SPSS in our study to evaluate neuroanatomical changes in our patient group at different time points and between patients and volunteers at the same time point. This approach treated each neuroanatomical measure from each wrist as a separate observation that was adjusted for within participant correlations. Subjects were treated as random effects, so the analysis was adjusted to each wrist’s own neuroanatomical levels.

There are some deficiencies in the present study. First, it was a single institutional study, as reflected in the small sample size. Second, the EDT and ultrasound examinations were not blinded to the clinical history, which could cause expectation bias. We need to perform further multisite, blinded clinical trials to revaluate our conclusions. Furthermore, we only enrolled severe idiopathic CTS patients in our study. Mild or moderate idiopathic CTS patients and secondary CTS patients should be enrolled in the follow-up experiments.

## Conclusions

Our study showed that neuroanatomical parameters were gradually improved after ECTR during one-year follow-up. The best time for US follow up is at 3-month postoperative time point for patients who do not show clinical improvement, since at this time the change is the greatest for most CTS patients. High-frequency ultrasonography is a valuable method in neuroanatomical measures to quantify morphological recovery of the median nerve after ECTR.

## Abbreviations

ADM: Abductor digiti minimi muscle
APB: Abductor pollicis brevis muscle
AUC: Area under the curve
CMAP: Compound muscle action potentials
CSA: Cross-sectional area
CTS: Carpal tunnel syndrome
DML: Distal motor latency
DSL: Distal sensory latency
ECTR: Endoscopic carpal tunnel release
EDT: Electrodiagnostic
EL: Edema length
EMG: Electromyography
MNCV: Motor nerve conduction velocities
NCSs: Nerve conduction studies
OCTR: Open carpal tunnel release
SNAPS: Sensory nerve action potential
SNCV: Sensory nerve conduction velocities
TCL: Transverse carpal ligament
