# Correction to: Sonographic follow-up after endoscopic carpal tunnel release for severe carpal tunnel syndrome: a one-year neuroanatomical prospective observational study

**DOI:** 10.1186/s12891-020-03555-0

**Published:** 2020-08-19

**Authors:** Miao Li, Jue Jiang, Qi Zhou, Chen Zhang

**Affiliations:** 1 Department of Ultrasound, the Second Affiliated Hospital of Xi’an Jiaotong University, Xi’an, Shaanxi 710004 People’s Republic of China; 2 The First Department of Orthopaedics, The Second Affiliated Hospital of Xi’an Jiaotong University, Xi’an, Shaanxi 710004 People’s Republic of China

**Correction to: BMC Musculoskelet Disord 20, 157 (2019)**

**https://doi.org/10.1186/s12891-019-2548-6**

Following publication of this article [1], the authors report the following Corrections to the main text:

i) The authors conducted both studies using data from the same patients. The authors did not reference the related Chinese language article [2]. This citation has been added.
ii) The main differences between the two publications were as followed: First, the two publications did not use the same design. The English language article [1] was designed in early 2014. It was approved by the Ethics Committee of Xi’an Jiaotong university medical college in June 2014 (NO. 2014–003). The period of patient enrollment in this study was from Dec 2014 to Oct 2016. The retrospective study published in Chinese-language article [2] was designed in early 2016 and approved by the Ethics Committee of the second affiliated hospital of Xi’an Jiaotong university in February 2016 (NO.2016–227). The patients in this study were retrospectively analyzed from Dec 2014 to Jan 2016. Second, the statistical analysis between the two articles were different also. Linear mixed model analysis was performed in the English language article [1] and paired *t*-test was used in Chinese language article [2].

Both the original article [1] and the Chinese language article [2] have been updated.
